# Berberine Alleviates Doxorubicin-Induced Myocardial Injury and Fibrosis by Eliminating Oxidative Stress and Mitochondrial Damage via Promoting Nrf-2 Pathway Activation

**DOI:** 10.3390/ijms24043257

**Published:** 2023-02-07

**Authors:** Yiyang Wang, Jia Liao, Yuanliang Luo, Mengsi Li, Xingyu Su, Bo Yu, Jiashuo Teng, Huadong Wang, Xiuxiu Lv

**Affiliations:** Department of Pathophysiology, School of Medicine, Jinan University, Guangzhou 510632, China

**Keywords:** berberine, doxorubicin, myocardial injury, fibrosis, Nrf2, oxidative stress

## Abstract

Doxorubicin (DOX)-related cardiotoxicity has been recognized as a serious complication of cancer chemotherapy. Effective targeted strategies for myocardial protection in addition to DOX treatment are urgently needed. The purpose of this paper was to determine the therapeutic effect of berberine (Ber) on DOX-triggered cardiomyopathy and explore the underlying mechanism. Our data showed that Ber markedly prevented cardiac diastolic dysfunction and fibrosis, reduced cardiac malondialdehyde (MDA) level and increased antioxidant superoxide dismutase (SOD) activity in DOX-treated rats. Moreover, Ber effectively rescued the DOX-induced production of reactive oxygen species (ROS) and MDA, mitochondrial morphological damage and membrane potential loss in neonatal rat cardiac myocytes and fibroblasts. This effect was mediated by increases in the nuclear accumulation of nuclear erythroid factor 2-related factor 2 (Nrf2) and levels of heme oxygenase-1 (HO-1) and mitochondrial transcription factor A (TFAM). We also found that Ber suppressed the differentiation of cardiac fibroblasts (CFs) into myofibroblasts, as indicated by decreased expression of α-smooth muscle actin (α-SMA), collagen I and collagen III in DOX-treated CFs. Pretreatment with Ber inhibited ROS and MDA production and increased SOD activity and the mitochondrial membrane potential in DOX-challenged CFs. Further investigation indicated that the Nrf2 inhibitor trigonelline reversed the protective effect of Ber on both cardiomyocytes and CFs after DOX stimulation. Taken together, these findings demonstrated that Ber effectively alleviated DOX-induced oxidative stress and mitochondrial damage by activating the Nrf2-mediated pathway, thereby leading to the prevention of myocardial injury and fibrosis. The current study suggests that Ber is a potential therapeutic agent for DOX-induced cardiotoxicity that exerts its effects by activating Nrf2.

## 1. Introduction

Doxorubicin (DOX) is an anticancer drug that has been widely used in treating several solid tumors [1,2]. Unfortunately, DOX treatment is associated with adverse effects such as cardiomyopathy and heart failure [3]. This cardiotoxicity is frequently detected years after the cessation of chemotherapy and cannot be effectively treated [4,5]. Although lowering the dose of DOX could reduce DOX-induced cardiotoxicity, this would lead to lower success rates of chemotherapy, which creates a dilemma for oncologists and cardiologists [6,7]. Dexrazoxane is currently the only cardioprotective agent approved by the United States Food and Drug Administration for use in patients receiving anthracycline chemotherapy. However, it can induce bone marrow suppression, liver toxicity and the development of secondary malignant tumors [8]. Therefore, it is of clinical importance to explore safe and effective adjuvant therapies for DOX-induced cardiotoxicity.

The molecular mechanism of DOX-induced cardiotoxicity has been extensively investigated. Although many studies have revealed that this condition may be related to inflammation, topoisomerase 2β, Ca^2+^ handling abnormalities and autophagy dysfunction [9,10,11], oxidative stress has been described to be most commonly associated with the complex pathophysiology of DOX-induced cardiotoxicity [12,13,14]. Oxidative stress is defined as the production of excessive reactive oxygen species (ROS) that cannot be quenched by the antioxidant defense system and may contribute to the pathophysiology of cardiac remodeling and heart failure [15]. Excessive ROS lead to oxidative damage to biological macromolecules and disrupt the integrity and function of the cell membrane, further leading to mitochondrial dysfunction and eventual cardiomyocyte apoptosis [16]. Oxidative stress, which is considered to be an important manifestation of DOX-induced myocardial remodeling [17], is also implicated in the pathogenesis of cardiac fibrosis both directly and via its involvement in cytokine and growth-factor signaling [18,19]. Cardiomyocytes are more susceptible to ROS because there are lower levels of antioxidant enzymes in the heart than in other organs [20]. Therefore, improving the endogenous cardiac antioxidant defense system is an attractive strategy to prevent DOX-induced cardiotoxicity.

Nuclear factor erythroid 2-related factor 2 (Nrf2), a redox-sensitive transcription factor, is the central regulator of the antioxidative stress defense system [21]. In response to oxidants, Nrf2 translocates to the nucleus, where it binds to antioxidant responsive elements to activate the transcription of downstream genes encoding cytoprotective enzymes such as heme oxygenase-1 (HO-1) [22]. Although aberrant HO-1 upregulation has been reported in several diseases such as Alzheimer disease and cancers [23,24], HO-1 protects against oxidative injury, regulates apoptosis, and modulates inflammation in normal cells [25,26]. It has been reported that activation of Nrf2/HO-1 signaling favors the enhancement of cell survival upon chemotherapy [27]. The phytochemicals such as curcumin [28] and formononetin [29] induce Nrf2/HO-1 signaling in reducing toxicity of oxaliplatin against liver and brain cells. These studies clearly demonstrate that Nrf2 signaling is of importance for alleviation of chemotherapy-mediated side effects. Furthermore, Nrf2 activates mitochondrial transcription factor A (TFAM), which leads to increased mitochondrial protein synthesis and mitochondrial mass, improved mitochondrial respiration, and depolarization of the mitochondrial membrane potential [30]. Activation of the Nrf2 pathway may attenuate DOX-induced oxidative stress and cardiac damage.

Berberine (Ber) is an isoquinoline alkaloid originally isolated from Chinese goldthread (*Coptis chinensis*) and possesses a wide range of biological activities, including antitumor, anti-inflammatory, cardiovascular-protective, and antioxidant activities, allowing it to reduce ROS production in various tissues [31,32,33]. Ber has been found to protect against oxidative stress injury in neuronal cells by enhancing Nrf2/HO-1 signaling [34]. We previously showed that Ber inhibits DOX-induced cardiomyocyte apoptosis in vitro and in vivo [35]; however, its mechanisms need to be explored in depth. We hypothesize that Ber may play an antioxidant role by promoting the activation of the Nrf2 signaling pathway, resulting in the prevention of DOX-induced cardiotoxicity. In this study, we aimed to investigate whether Ber could protect against DOX-induced myocardial injury and fibrosis by preventing oxidative stress and mitochondrial damage via regulation of the Nrf2-mediated pathway.

## 2. Results

### 2.1. The Effect of Ber on Preventing DOX-Induced Cardiac Diastolic Dysfunction and Fibrosis Is Associated with Alleviating Oxidative Stress

**Figure 1:**
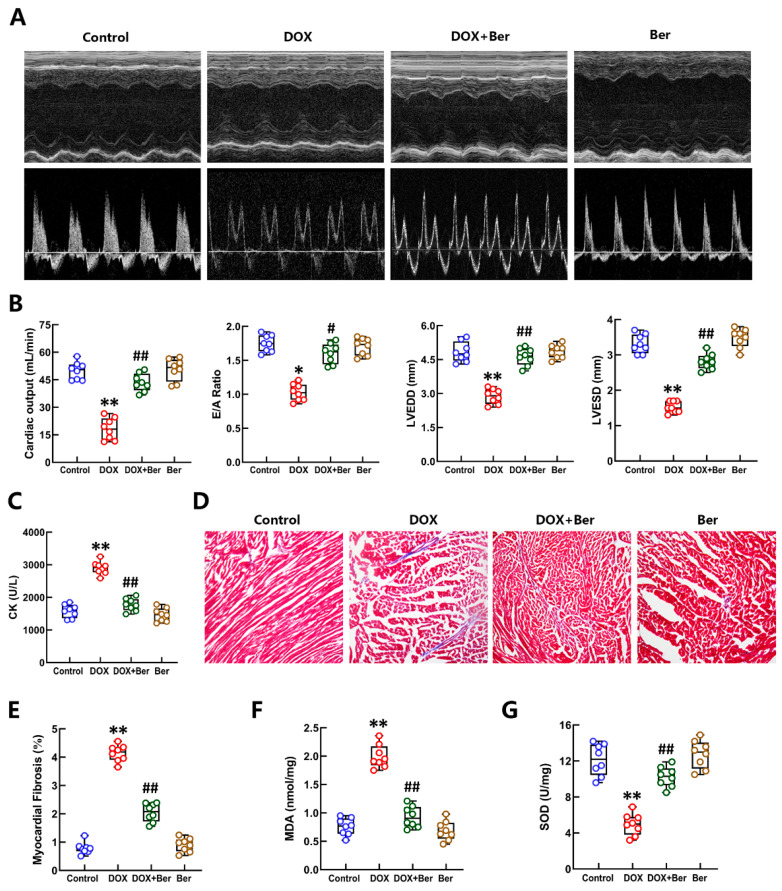
The alleviating effects of Ber on DOX-induced cardiotoxicity. (**A**) Representative cardiac M-mode (upper row) and Doppler wave mode (bottom row) echocardiograms obtained from rats treated with normal saline or DOX with/without Ber. (**B**) Quantitative group data for echocardiographic measurements (cardiac output, the E/A ratio, LVEDD and LVESD). (**C**) The level of creatine kinase (CK) in the sera of rats. (**D**) Representative 200× images of myocardial tissue sections stained with Masson’s trichrome. (**E**) Quantification of the fractional area of cardiac fibrosis based on five randomly selected fields of stained myocardial tissue sections. (**F**) The MDA content in the cardiac tissue of rats. (**G**) The activity of SOD in cardiac tissue. Mean ± standard error of the mean (*n* = 8). * *p* < 0.05 and ** *p* < 0.01 versus the control group. # *p* < 0.05 and ## *p* < 0.01 versus the DOX group.

Two days after the last administration, echocardiography was performed to study the effects of Ber and/or DOX treatment on left ventricular function. Echocardiographic examination demonstrated that DOX treatment markedly reduced CO and the E/A ratio, an index used to reflect diastolic function. In contrast, CO and the E/A ratio were higher in the Ber (60 mg/kg) + DOX group than in the DOX group. Moreover, LVEDD and LVESD decreased after DOX challenge. LVEDD and LVESD were increased in the Ber-pretreated group compared with the DOX group (Figure 1A,B). Usually, cardiac injury is accompanied by the release of enzymes such as creatine kinase (CK) into the blood [36]. In our study, as shown in Figure 1C, DOX increased serum CK levels from 1378.8 ± 225.1 U to 2640 ± 443.8 U. Pretreatment with Ber markedly reduced the elevated CK content to 1586.5 ± 170.8 U. To further study the mechanism of Ber against DOX-initiated myocardial injury, cardiac fibrosis in rats treated with DOX and/or Ber was detected by Masson’s trichrome staining. The results of Masson’s staining in the four groups are shown in Figure 1D,E. After Masson’s staining, cardiomyocytes were stained red, whereas collagen fibers were stained blue. More collagen deposition was observed in the hearts of rats administered DOX than in the hearts of control rats. The ratio of collagen area to total area was measured. The fractional area of cardiac fibrosis was significantly larger in the DOX group than in the control group. Fibrosis of the heart was markedly reduced in the DOX + Ber group compared with the DOX group. Furthermore, we measured MDA and SOD levels in the myocardium of rats treated with DOX with or without Ber. The MDA content in cardiac tissue was increased from 0.92 ± 0.025 nmol/mg protein to 1.54 ± 0.01 nmol/mg protein after DOX injection. However, the SOD level in cardiac tissues was decreased from 12.28 ± 0.61 U/mg to 6.23 ± 0.47 U/mg. Ber obviously decreased the elevated MDA content to 1.44 ± 0.028 nmol/mg and increased the levels of SOD to 10.64 ± 0.88 nmol/mg protein. This suggests that the effect of Ber in protecting against DOX-induced cardiac diastolic dysfunction and fibrosis is related to inhibiting oxidative stress.

### 2.2. Ber Inhibited Cardiomyocyte Apoptosis, Oxidative Stress, Mitochondrial Injury and Activated Nrf-2-Mediated Signaling Pathway in Cardiomyocytes after DOX Stimulation

**Figure 2:**
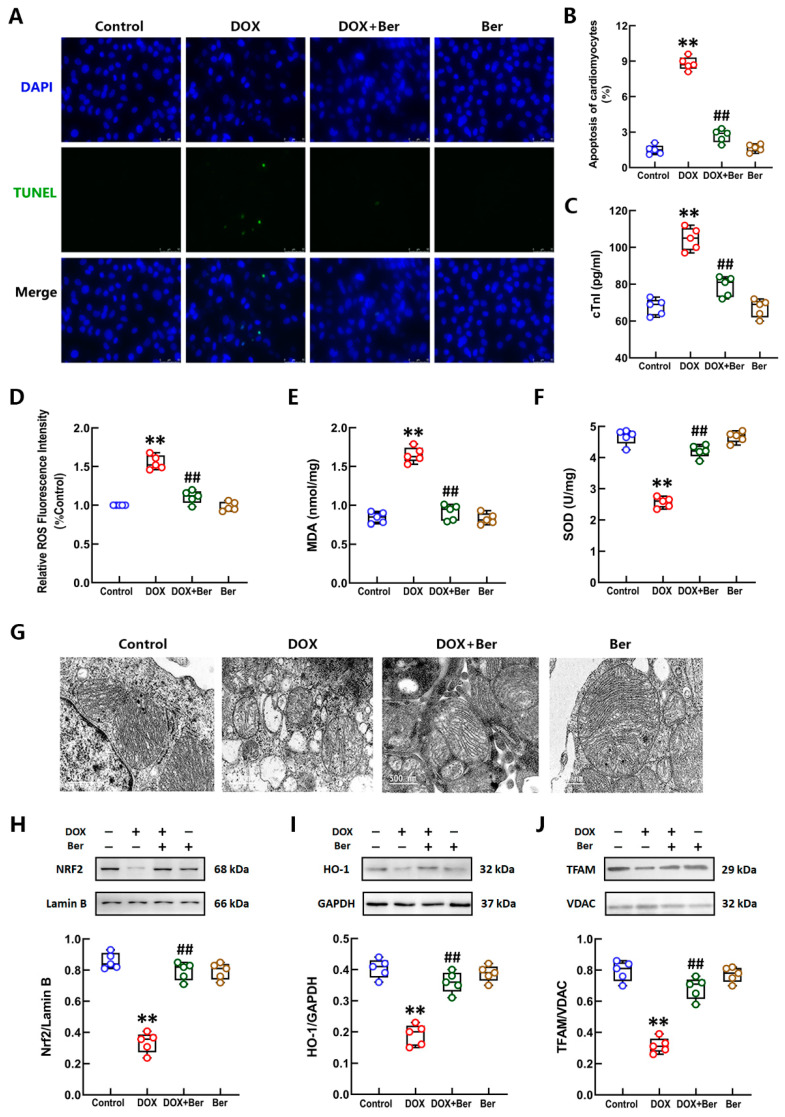
Ber suppressed DOX-induced cardiomyocyte injury, mitochondrial damage and oxidative stress. (**A**) Representative photomicrographs of TUNEL staining (green). Nuclei were stained with DAPI (blue). The scale bar represents 50 μm. (**B**) The rate of apoptosis was expressed as the ratio of TUNEL-positive cardiomyocyte nuclei (green) to total number of cardiomyocyte nuclei (blue). The numbers of apoptotic cells were counted in five randomly selected fields for each TUNEL-stained sample. (**C**) The release of cTnI from myocardiocytes. (**D**–**F**) The relative levels of ROS (**D**), MDA (**E**) and SOD (**F**) in myocardiocytes stimulated with DOX and/or Ber. (**G**) Mitochondrial morphology of cardiomyocytes was observed by transmission electron microscopy. The scale bar represents 500 nm. (**H**–**J**) The levels of nuclear Nrf2 (**H**), cytoplasmic HO-1 (**I**) and mitochondrial TFAM (**J**) in cardiomyocytes treated with DOX with/without Ber. Quantitative analysis of the relative protein levels is shown at the bottom of the images. Mean ± standard error of the mean (*n* = 5). ** *p* < 0.01 versus the control group. ## *p* < 0.01 versus the DOX group.

We found that Ber inhibits DOX-induced cardiomyocyte apoptosis in vitro and in vivo in our previous study [35], but the mechanisms involved have not been thoroughly explored. Here, the TUNEL assay results again confirmed that Ber reduced the apoptosis of neonatal rat cardiomyocytes after DOX administration (Figure 2A,B). The assessment of DOX-induced cytotoxicity in cardiomyocytes was performed using the cTnI test with a culture medium of neonatal rat cardiomyocytes. The cells were preincubated with Ber for 30 min and treated with DOX for 24 h. The results of the cTnI release assay showed that DOX significantly increased cTnI content in the culture medium of cardiomyocytes. Pretreatment with Ber considerably decreased cTnI content in the culture medium of cardiomyocytes treated with DOX (Figure 2C). To further ascertain the effects of Ber on DOX-induced oxidative stress in neonatal rat cardiomyocytes, we examined ROS production and MDA and SOD levels in cardiomyocytes. Consistent with the results of the in vivo experiments, as shown in Figure 2D–F, DOX increased ROS production and MDA content but decreased SOD levels in cardiomyocytes, and this effect was reversed by Ber. It is well known that mitochondria have important physiological functions, such as oxidative phosphorylation, electron transfer and energy metabolism, and are the source of intracellular oxidative stress [37]. Many studies have found that DOX induces the production of excess ROS in mitochondria and leads to mitochondrial dysfunction [38,39]. We previously found that exposure of neonatal rat cardiomyocytes to DOX induces a marked decrease in the mitochondrial membrane potential, an effect that is reduced by Ber [35]. In the current research, we examined the changes in mitochondrial morphology in DOX- and/or Ber-treated cardiomyocytes using transmission electron microscopy. As shown in Figure 2G, mitochondria in the control group were regular with distinct cristae. After treatment with DOX for 2 h, the mitochondrial structure was not distinct, the mitochondrial cristae were swollen or disordered, and vacuolization was visible. We also observed partial mitochondrial membrane rupture. Interestingly, irregular mitochondria, vacuolization, and partial mitochondrial membrane rupture were alleviated in the Ber pretreatment group. The changes in mitochondrial ultrastructure indicate that Ber notably reduced DOX-induced mitochondrial damage in cardiomyocytes. To clarify the mechanism underlying the protective effects of Ber against DOX-induced oxidative stress and mitochondrial damage, we examined the protein levels of nuclear/cytosolic Nrf2, HO-1, and the mitochondrial marker TFAM in cardiomyocytes 2 h after DOX treatment. The Western blot results showed that DOX induced a modest increase in the levels of nuclear Nrf2 protein in neonatal rat cardiomyocytes. Compared with the DOX treatment, Ber pretreatment elevated nuclear Nrf2 levels in cells. Additionally, the protein levels of HO-1 and TFAM were significantly lower in DOX-treated cells than in control cells, and this effect that was partially reversed by Ber pretreatment (Figure 2H–J). These results demonstrate that the protective effect of Ber against DOX-induced cardiomyocyte injury is most likely related to the complete inhibition of oxidative stress and mitochondrial damage through activation of the Nrf2-mediated pathway.

### 2.3. The Effect of Ber on Protecting Cardiomyocytes against DOX-Induced Injury, Mitochondrial Damage and Oxidative Stress Was Dependent on Nrf2 Activation

**Figure 3:**
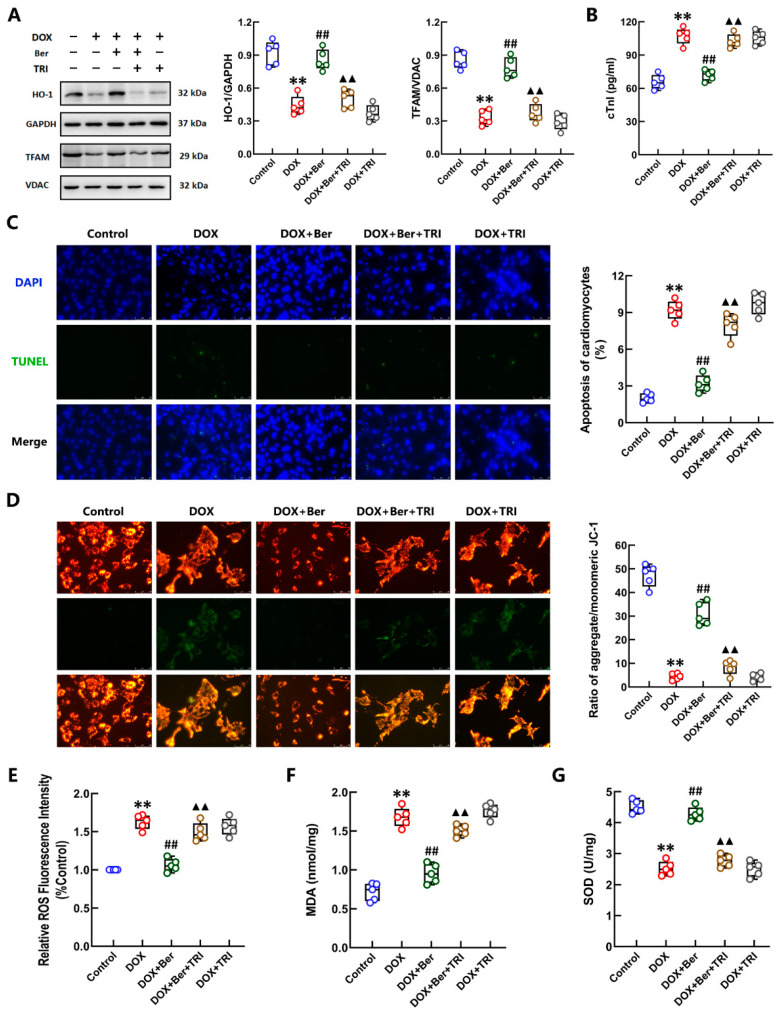
The inhibitor of NRF2 suppressed the protective effect of Ber on cardiomyocytes. Cardiomyocytes were treated with the NRF2 inhibitor trigonelline (TRI) 30 min prior to DOX and Ber stimulation. (**A**) The levels of cytoplasmic HO-1 and mitochondrial TFAM in cardiomyocytes were measured by Western blotting. Quantitative analysis of the relative protein levels is shown on the right-hand side of the images. (**B**) The release of cTnI from cardiomyocytes. (**C**) Representative photomicrographs TUNEL-stained sections (**left**). The rate of apoptosis was expressed as the ratio of TUNEL-positive cardiomyocyte nuclei (green) to total number of cardiomyocyte nuclei (blue). The numbers of apoptotic cells were counted in five randomly selected fields for each sample (**right**). The scale bar represents 50 μm. (**D**) Representative images of JC-1 fluorescence (**left**) and the ratio of aggregated to monomeric JC-1 (**right**). The mitochondrial membrane potential in cardiomyocytes was visualized by JC-1 staining. Green fluorescence indicates monomeric JC-1 (low potential), and red fluorescence represents aggregated JC-1 (high potential). The scale bar represents 50 μm. (**E**–**G**) The relative levels of ROS (**E**), MDA (**F**) and SOD (**G**) in cardiomyocytes. Mean ± standard error of the mean (*n* = 5). ** *p* < 0.01 versus the control group. ## *p* < 0.01 versus the DOX group. ▲▲ *p* < 0.01 versus the DOX + Ber group.

To confirm the role of Nrf2 activation in the mechanism underlying the protective effect of Ber against DOX-induced cardiomyocyte apoptosis and injury, we treated cardiomyocytes with an inhibitor of Nrf2, trigonelline (TRI), 30 min prior to Ber and DOX stimulation. As depicted in Figure 3A, the Western blot results showed that the effect of Ber in increasing HO-1 and TFAM levels in DOX-treated cardiomyocytes was completely blocked by TRI. Moreover, inhibition of Nrf2 activation with 1 μM TRI reversed the inhibitory effect of Ber on cardiomyocyte apoptosis and the increase in cTnI levels induced by DOX but did not affect apoptosis or cTnI levels in cardiomyocytes treated with DOX alone (Figure 3B,C). Pretreatment with TRI also suppressed the effect of Ber in maintaining the mitochondrial membrane potential (MMP) of cardiomyocytes stimulated with DOX but had no effect on cardiomyocytes challenged with DOX alone (Figure 3D). Furthermore, we confirmed the role of Nrf2 activation in the protective effect of Ber against DOX-initiated oxidative stress in cardiomyocytes. ROS and MDA levels showed a marked decrease in cardiomyocytes costimulated with Ber and DOX for 24 h compared with those treated with DOX alone. TRI reversed the inhibitory effects of Ber on DOX-induced increases in ROS and MDA levels (Figure 3E,F). In addition, the SOD content was obviously increased in the DOX + Ber + TRI group compared with the DOX- and Ber-treated cardiomyocytes not pretreated with TRI (Figure 3G). The administration of TRI did not markedly alter the levels of HO-1, ROS, MDA or SOD in cardiomyocytes stimulated by DOX alone. These findings demonstrate that the suppressive effect of Ber on DOX-induced cardiomyocyte injury, mitochondrial damage and oxidative stress was dependent on Nrf2 activation.

### 2.4. Ber Suppressed DOX-Induced Differentiation of Cardiac Fibroblasts (CFs) into Myofibroblasts

**Figure 4:**
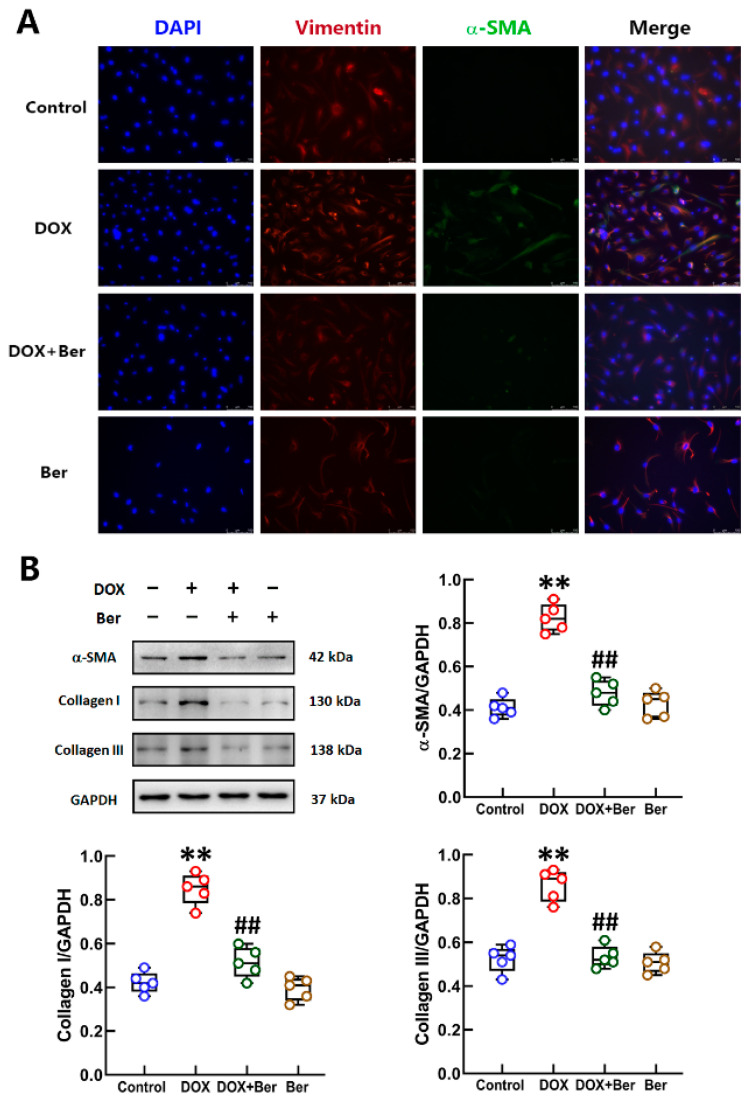
Ber inhibited cardiac fibroblast (CF) differentiation and collagen I/III synthesis after DOX stimulation. (**A**) Representative immunofluorescence images of CFs after DOX and/or Ber stimulation for 24 h. α-SMA (red)-labeled myofibroblasts, vimentin (green)-labeled CFs, and DAPI (blue)-stained nuclei are shown. The scale bar represents 50 μm. (**B**) The protein levels of α-SMA, collagen I and collagen III in CFs treated with DOX and/or Ber for 24 h were measured by Western blotting. Quantitative analysis of the relative protein levels is shown in the panels next to the images. Mean ± standard error of the mean (*n* = 5). ** *p* < 0.01 versus the control group. ## *p* < 0.01 versus the DOX group.

Cardiac fibrosis was observed after DOX treatment, suggesting that DOX is a potent inducer of fibroblast-to-myofibroblast differentiation characterized by high expression of α-smooth muscle actin (α-SMA) [40]. To determine the effect of DOX and/or Ber on the differentiation of CFs into myofibroblasts, first, the level of α-SMA in DOX-administered CFs with or without Ber was assessed by immunofluorescence staining. As expected, compared with control CFs, the CFs treated with DOX for 24 h exhibited an obvious increase in α-SMA expression. We observed a drastic decrease in α-SMA levels in the DOX + Ber-treated CFs compared with the CFs treated with DOX alone. Ber slightly increased the expression of α-SMA in CFs not stimulated with DOX, but the increase was not statistically significant (Figure 4A). The Western blot results showed that the protein expression levels of α-SMA, collagen I and collagen III in DOX-treated cells were significantly increased compared with those in control cells. Ber completely inhibited the DOX-induced increases in α-SMA, collagen I and collagen III levels in CFs (Figure 4B). This means that treatment with Ber prior to DOX administration inhibits the differentiation of CFs.

### 2.5. Ber Alleviated DOX-Induced Oxidative Stress and MMP Loss and Promoted Activation of the Nrf2 Pathway in CFs

**Figure 5:**
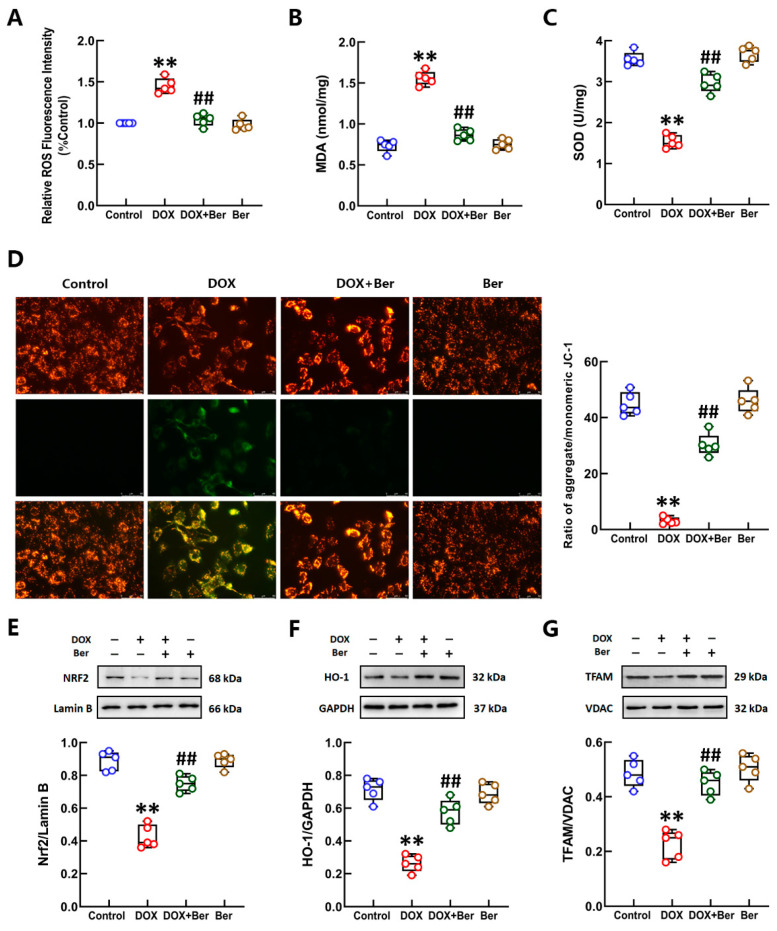
The effects of Ber on oxidative stress and the mitochondrial membrane potential in CFs after DOX administration were mediated by activation of the Nrf2-mediated pathway. (**A**–**C**) The relative levels of ROS (**A**), MDA (**B**) and SOD (**C**) in CFs treated with DOX and/or Ber. (**D**) Representative images of JC-1 fluorescence (**left**) and the ratio of aggregated to monomeric JC-1 (**right**). The mitochondrial membrane potential in CFs was visualized by JC-1 staining. Green fluorescence indicates monomeric JC-1, and red fluorescence represents aggregated JC-1. The scale bar represents 50 μm. (**E**–**G**) The levels of nuclear Nrf2 (**E**), cytoplasmic HO-1 (**F**) and mitochondrial TFAM (**G**) in CFs treated with DOX with/without Ber. Quantitative analysis of the relative protein levels is shown at the bottom of the images. Mean ± standard error of the mean (*n* = 5). ** *p* < 0.01 versus the control group. ## *p* < 0.01 versus the DOX group.

To evaluate the effect of Ber on oxidative stress in CFs stimulated with DOX, we measured the levels of ROS, MDA and SOD in CFs treated with DOX and/or Ber for 24 h. The results showed that DOX obviously increased the levels of ROS and MDA and decreased the SOD content in the CFs. Similar to the results in cardiomyocytes, Ber significantly inhibited DOX-induced alterations in ROS, MDA and SOD levels in CFs (Figure 5A–C). We further determined the effect of Ber on the MMP in DOX-treated CFs stained with JC-1. When the MMP is normal, JC-1 forms J-aggregates in the mitochondrial matrix, exhibiting red fluorescence. Conversely, JC-1 exists as a monomer that fluoresces green when the MMP is lost. Thus, a change in florescence from red to green indicates a decrease in the MMP. As shown in Figure 5D, the ratio of red fluorescence intensity to green fluorescence intensity was markedly lower in the group treated with DOX for 12 h than in the control group. In contrast, the intensity of red/green fluorescence decreased in DOX-stimulated CFs pretreated with Ber. As determined by Western blotting, Ber markedly increased the levels of Nrf2, HO-1 and TFAM in CFs 2 h after DOX stimulation (Figure 5E–G). These findings indicate that Ber promotes the activation of the Nrf2 signaling pathway, which may alleviate DOX-mediated CF differentiation by suppressing oxidative stress and mitochondrial injury.

### 2.6. An Inhibitor of Nrf2 Abolished the Inhibitory Effect of Ber on CF Differentiation into Myofibroblasts Induced by DOX

**Figure 6:**
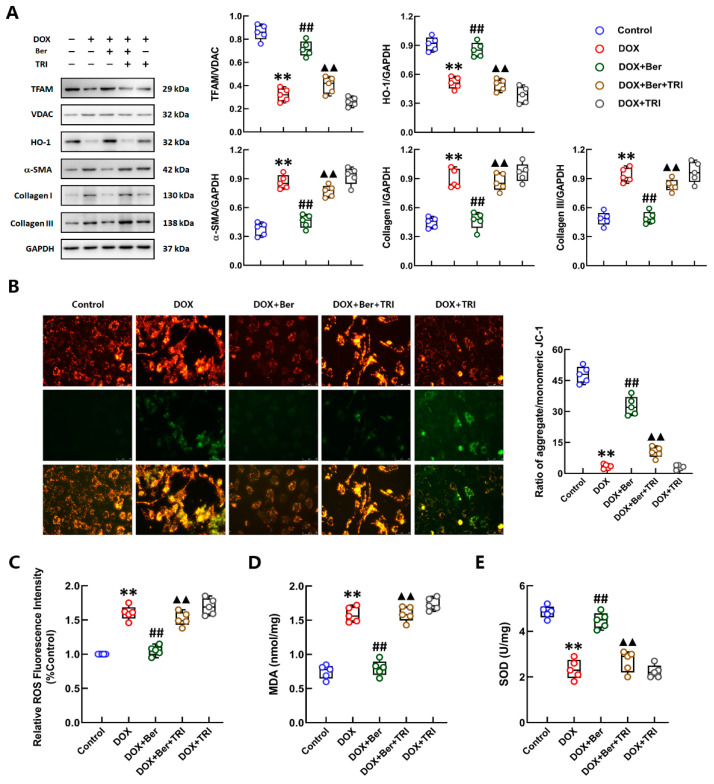
TRI reversed the effect of Ber on inhibiting CF differentiation and collagen I/III synthesis via inhibition of the Nrf2 signaling pathway. CFs were treated with TRI 30 min prior to DOX and Ber administration. (**A**) The expression levels of TFAM, HO-1, α-SMA, collagen I and collagen III in CFs treated with DOX and/or Ber for 24 h were measured by Western blotting. Quantitative analysis of the relative protein levels is shown in the panels next to the images. (**B**) Representative images of JC-1 fluorescence (**left**) and the ratio of aggregated to monomeric JC-1 (**right**). Green fluorescence indicates monomeric JC-1, and red fluorescence represents aggregated JC-1. The scale bar represents 50 μm. (**C**–**E**) The relative levels of ROS (**C**), MDA (**D**) and SOD (**E**) in the CFs. Mean ± standard error of the mean (*n* = 5). ** *p* < 0.01 versus the control. ## *p* < 0.01 versus the DOX group. ▲▲ *p* < 0.01 versus the DOX + Ber group.

Furthermore, we explored whether the effect of Ber in inhibiting DOX-initiated CF differentiation was achieved by activation of Nrf2. CFs were preincubated with TRI for 30 min before treatment with LPS and DOX. The Western blot results are shown in Figure 6A, Ber upregulated the expression of HO-1 and TFAM and reduced α-SMA and collagen I/III expression in DOX-challenged CFs. These effects of Ber were reversed by TRI. Stimulation with TRI had no effect on the differentiation of DOX-treated CFs. We also tested the effect of TRI on the MMP and oxidative stress in CFs after DOX and Ber treatment. Consistent with the results of the cardiomyocyte experiments, the inhibitory action of Ber on DOX-induced MMP loss in CFs was markedly prevented by TRI (Figure 6B). Moreover, TRI completely inhibited the effect of Ber on the downregulation of ROS and MDA and the increase in SOD levels in CFs after DOX stimulation (Figure 6C–E). These data indicate that the effect of Ber in preventing DOX-induced differentiation of CFs is mediated by activation of the Nrf2 signaling pathway, which suppresses oxidative stress and mitochondrial injury in CFs.

## 3. Discussion

**Figure 7:**
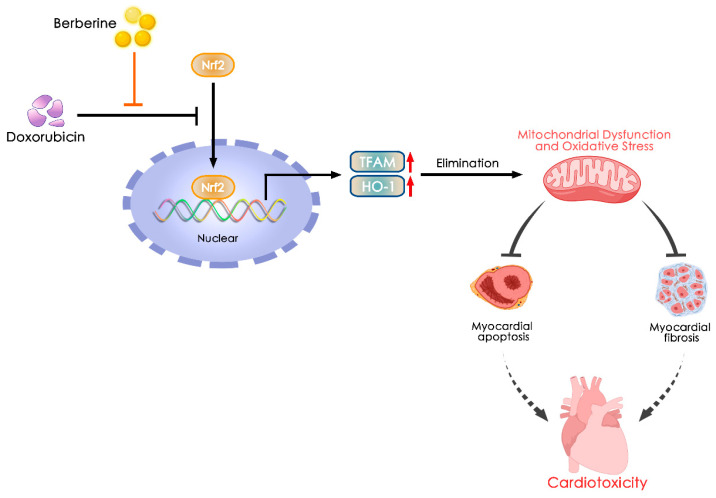
Proposed signaling mechanisms by which Ber inhibits DOX-induced cardiotoxicity. The effects of Ber on suppressing DOX-induced myocardial injury and fibrosis are mediated by inhibition of mitochondrial dysfunction and oxidative stress via activation of the Nrf2-mediated signaling pathway.

Although Doxorubicin is an effective chemotherapeutic drug for treating multiple cancers worldwide, the risk of DOX-induced cardiotoxicity, such as myocyte destruction, left ventricular dysfunction and cardiac remodeling, has been noted [41,42]. The pathological mechanisms of DOX-induced cardiac injury are complicated and have not been fully elucidated. It has been suggested that increased oxidative stress, such as the production of ROS, and compromise of the antioxidant system play pivotal roles in the disturbance of cardiac homeostasis in DOX-induced cardiotoxicity [43,44]. Mitochondria are the main sites of ROS generation and the key targets of DOX [45]. Therefore, exploring ways to inhibit oxidative stress and protect mitochondria is an attractive approach to reduce cardiac injury after DOX treatment. Ber hydrochloride is an effective antioxidant and free radical scavenger that prevents ROS formation [46]. In this research, we identified Ber as a potential therapeutic drug in treating DOX-induced cardiotoxicity, as indicated by its ability to inhibit myocardial injury and fibrosis and improve cardiac diastolic function. Mechanistically, we revealed that Ber completely inhibited oxidative stress and mitochondrial injury by inducing Nrf2-mediated upregulation of HO-1 and TFAM expression in cardiomyocytes and CFs, thereby leading to reduced cardiomyocyte apoptosis and CF differentiation; these effects were responsible for the beneficial roles of Ber in DOX-induced cardiotoxicity (Figure 7). These results indicate that Ber may be a potential therapeutic drug for the prevention of DOX-induced heart failure.

The evidence shows that left ventricular dysfunction and cardiac fibrosis have crucial roles in the pathogenesis of DOX-induced cardiotoxicity [47,48]. Our previous research showed that DOX and/or Ber have no obvious effect on left ventricular ejection fraction and fractional shortening, suggesting that DOX may only cause mild impairment of left ventricular systolic function. In this study, we confirmed that DOX reduced CO, the E/A ratio, LVEDD, and LVESD, and these effects were reversed by pretreatment with Ber. This means that DOX treatment caused obvious impairment of cardiac diastolic function, as reflected by a decreased E/A ratio, and this effect was significantly prevented by Ber. CK is one of the most important markers of cardiotoxicity. In this study, Ber markedly inhibited the increase in serum CK activity induced by DOX. In addition, our results further confirmed that Ber suppressed DOX-induced cardiac fibrosis, as indicated by Masson’s staining. Oxidative stress causes damage to the lipid membrane and other cellular components due to an imbalance between the oxidant and antioxidant enzyme systems, which may be an important factor in DOX-induced cardiotoxicity [49]. Several studies have reported that DOX tends to induce the generation of ROS during metabolism [50,51]. Compared with other tissues, the myocardium is more vulnerable to oxidative stress, possibly due to low levels of antioxidant enzymes that scavenge ROS [52,53]. Consistent with previous studies, our results confirmed that DOX promoted ROS production in the myocardium of rats. As high levels of ROS cause lipid peroxidation, we measured MDA content and the activity of SOD, a major antioxidant enzyme. We found that Ber decreased the production of MDA and increased SOD levels after DOX challenge in rat cardiac tissue. Based on the above results, we conclude that Ber alleviated DOX-induced myocardial oxidative stress damage, which may be an important mechanism underlying its protective effect against cardiac diastolic dysfunction and fibrosis.

Some studies have suggested that cardiomyocyte apoptosis is associated with irreversible heart failure. We previously found that Ber protects against DOX-induced cardiomyocyte apoptosis through the mitochondria-mediated apoptotic pathway. In this study, we more deeply explored the mechanisms by which Ber protects cardiomyocytes against DOX-induced injury in vitro. The results showed that 1 μM Ber not only inhibited cardiomyocyte apoptosis but also decreased ROS and MDA production and reduced SOD levels in cardiomyocytes treated with DOX. SOD is the first line of defense against oxygen-derived free radicals, and mitochondria are rich in SOD and are the source of intracellular ROS [54]. DOX is retained in the inner mitochondrial membrane during complexation with cardiolipin [55]. Given that Ber mainly enhanced SOD activity and reduced ROS production, we speculated that the key players in the effects of Ber on DOX-induced oxidative stress in cardiomyocytes may be mitochondria. Therefore, we examined the integrity of mitochondria in primary cultured cardiomyocytes. Interestingly, we observed that the morphology of mitochondria was markedly changed after 2 h of DOX treatment. TEM showed that Ber reduced the swelling of mitochondrial cristae and the disorder and rupture of mitochondria induced by DOX. Combined with our previous finding that Ber reverses DOX-induced MMP loss in cardiomyocytes, these findings indicate that Ber plays an important role in maintaining mitochondrial integrity in cardiomyocytes upon DOX stimulation.

CFs, the most abundant interstitial cells in the adult mammalian heart, are a key source of extracellular matrix proteins, such as collagen I and III, which surround myocytes [56]. Under pathological conditions, injurious factors lead to CF differentiation into cardiac myofibroblasts, which highly express α-SMA and produce more collagen I and III, which contribute to the development of cardiac fibrosis [57]. Cardiac compliance and diastolic function decline accompanied by aggravation of myocardial fibrosis, ultimately leading to cardiac insufficiency and even heart failure [58]. In this study, CFs differentiated into myofibroblasts after DOX treatment in vitro, which is consistent with a previous report [19]. Interestingly, Ber abolished DOX-induced CF differentiation, as indicated by the downregulation of α-SMA and collagen I/III. Importantly, these effects of Ber were accompanied by alleviated MMP loss and decreased intracellular oxidative stress, as indicated by ROS accumulation and reduced levels of the antioxidant enzyme SOD. These studies suggest that the inhibitory impact of Ber on the effects of DOX mediates the fibroblast–myofibroblast transition and is associated with complete inhibition of oxidative stress and maintenance of mitochondrial function.

Nrf2 is a transcription factor that plays a key role in cellular defense against oxidative stress by initiating the transcription of antioxidant genes, including HO-1 [59]. In addition, Nrf2 acts on many genes whose products are imported into mitochondria, such as TFAM [60]. TFAM is necessary for maintaining mitochondrial function because it protects mitochondrial DNA against ROS damage and has been found to play an important role in the Nrf2-mediated protective effect on mitochondrial bioenergetics and biogenesis [61,62]. Numerous studies support the notion that downregulation of Nrf2 is associated with various oxidative stress-related cardiomyopathies, such as DOX-induced cardiotoxicity [63,64,65]. However, some studies have shown that activation of the Nrf2 pathway accelerates ferroptosis and promotes DOX-induced cardiotoxicity [66,67]. This discrepancy indicates that the effect of Nrf2 on DOX-induced cardiotoxicity requires further investigation. The results of this study showed that DOX disrupted the nuclear translocation of Nrf2 in both cardiomyocytes and CFs, which was completely reversed by Ber. Activation of Nrf2 by Ber was found to be beneficial for preserving the intracellular activity of antioxidant enzymes and mitochondrial biogenesis during DOX stimulation due to an increase in HO-1 and TFAM protein synthesis. Moreover, we further confirmed that activated Nrf2 participates in the suppressive effects of Ber on DOX-induced cardiomyocyte injury and CF differentiation by using the Nrf2 inhibitor TRI. The data showed that TRI inhibited the Ber-mediated suppression of cardiomyocyte apoptosis, CF differentiation, MMP loss and oxidative stress in cardiomyocytes and CFs challenged with DOX. Furthermore, the Ber-induced upregulation of HO-1 and TFAM in DOX-treated cardiomyocytes and CFs was completely suppressed by Nrf2 inhibition. It has been reported that stimulation of Nrf2 signaling protects H9c2 cardiomyocytes from damage caused by DOX [68,69]. Pharmacological agents with naturally occurring compounds as the most common have been used for inducing Nrf2 signaling in DOX amelioration [70,71]. In this study, our findings provide strong evidence that Ber inhibits DOX-induced cardiomyocyte injury and CF differentiation by activating Nrf2 to alleviate oxidative stress and protect mitochondria. This study is the first to show that Ber inhibits chronic DOX-induced cardiotoxicity through activating the Nrf2-mediated pathway. However, further studies must be carried out in clinical trials to confirm how to use Ber in the treatment of DOX-triggered heart failure. Meanwhile, we only studied the effect of Ber on activation of Nrf2 in DOX-treated cardiomyocytes and CFs, and the mechanisms need to be studied in depth.

## 4. Materials & Methods

### 4.1. Materials

Doxorubicin hydrochloride (D1515) and berberine hemisulfate salt (B3412) were purchased from Sigma Aldrich (St. Louis, MO, USA). Trigonelline (HY-N0414) was obtained from MedChemExpress (Monmouth Junction, NJ, USA). Anti-α-SMA (ab7817), anti-Nrf2 (ab137550), anti-TFAM (ab131607), and anti-voltage-dependent anion channel (VDAC, ab15895) antibodies were obtained from Abcam (Cambridge, UK). Antibodies against vimentin (#5741), lamin B (#13435) and GAPDH (#2118) were purchased from Cell Signaling Technology, Inc. (Beverly, MA, USA). Antibodies against collagen I (WL0088) and collagen III (WL03186) were obtained from Wanleibio (Shenyang, Liaoning, China). Alexa Fluor 647-labeled goat anti-rabbit IgG (A32733), Alexa Fluor 488-labeled goat anti-mouse IgG (A11001), goat anti-rabbit IgG (#31462) and goat anti-mouse IgG (#31438) were obtained from Thermo Fisher Scientific (Logan, UT, USA). Reactive oxygen species (ROS, S0033S), malondialdehyde (MDA, S0131S) and superoxide dismutase (SOD, S0101S) assay kits were purchased from Beyotime Biotechnology (Nantong, China).

### 4.2. Animals and Treatment Procedures

Wild-type male Sprague-Dawley rats (8 to 10 weeks old) were obtained from the Medical Laboratory Animal Center of Guangdong Province (Guangzhou, China) and housed under standard conditions (12 h light/12 h dark cycle, 24 °C, and 50–70% humidity) with free access to food and water. The rats were randomly divided into four groups: the control group (received saline as vehicle); the DOX group (received 2.5 mg/kg DOX three times per week); the Ber group (treated with Ber at dose of 60 mg/kg via intragastric administration); and the DOX + Ber group (treated with Ber 30 min before each DOX injection). There were eight rats in each group. The rats were returned to their original cages after drug treatment and provided free access to food and water. After three weeks, the rats were anesthetized with diethyl ether and sacrificed. Then, the left ventricle of the heart was obtained for Western blotting and Masson’s staining. All animal experiments were conducted according to the guidelines for the Care and Use of Laboratory Animals of the National Institutes of Health and approved by the Animal Care and Use Committee at Jinan University School of Medicine.

### 4.3. Echocardiography

Two days after the last drug treatment, cardiac function was assessed with the VisualSonics^R^ Vevo 770^TM^ High-Resolution In Vivo Micro-Imaging System (VisualSonics, Inc., Toronto, ON, Canada), as described previously [72]. Rats were anesthetized with 1.5% isoflurane (Rhodia UK Ltd., Avonmouth, Bristol, UK) and imaged in the supine position using a 17.5-MHz-centered frequency RMV 707 scanhead. Two-dimensional B-mode and M-mode images were acquired by a technician who was blinded to the study design. The left ventricular end-diastolic diameter (LVEDD) and left ventricular end-systolic diameter (LVESD) were measured. The peak early filling (E wave) and late diastolic filling (A wave) velocities were measured on the M-mode parasternal short-axis tracing at the papillary muscle level. Cardiac output (CO) and the E/A ratio were calculated with Vevo770TM imaging system software, and data from at least three consecutive cardiac cycles were averaged.

### 4.4. Masson’s Trichrome Staining and Biochemical Analyses

After echocardiographic assessment, the rats were immediately anesthetized with pentobarbital sodium (50 mg/kg, intraperitoneally). An appropriate depth of anesthesia was confirmed by the disappearance of the corneal reflex, loss of the pedal reflex, and failure to respond to a skin incision. Cardiac tissues were fixed in 10% buffered formalin and embedded in paraffin. Paraffin-embedded cardiac tissues were cut into 5 μm-thick sections with standard techniques. For evaluation of cardiac fibrosis, the cardiac tissue sections were stained with Masson’s trichrome according to the manufacturer’s instructions and then examined under light microscopy. Fibrosis was assessed in 10 randomly selected fields per section at high magnification. ImageJ software (v1.53) was used to quantify the fractional area of cardiac fibrosis. Blood samples were collected in tubes and allowed to clot at room temperature. Serum was separated by centrifugation at 400× *g* for 15 min at 4 °C for the determination of creatine kinase (CK) activity.

### 4.5. Neonatal Rat Cardiomyocyte and Cardiac Fibroblast Culture and Cytotoxicity Assay

Neonatal Sprague-Dawley rats obtained from the Medical Laboratory Animal Center of Guangdong Province (Guangzhou, China) were deeply anesthetized with pentobarbital sodium (100 mg/kg). The hearts were excised, and left ventricular cardiomyocytes and fibroblasts were enzymatically dissociated. The digested cells were cultured in Dulbecco’s modified Eagle medium supplemented with 10% fetal bovine serum, 100 U/mL penicillin, and 100 µg/mL streptomycin at 37 °C for 48 h in a humidified atmosphere containing 5% CO_2_. The cells were treated with 1.0 μM Ber or vehicle for 20 min and then exposed to 1.0 μM DOX for the indicated period. Cardiomyocyte cytotoxicity was assessed by measuring the release of cardiac troponin I (cTnI, LifeSpan BioSciences, New York, NY, USA).

### 4.6. Terminal Deoxynucleotidyl Transferase dUTP Nick End Labeling (TUNEL) Assay

Cardiomyocyte apoptosis was determined using an in situ cell death detection kit (Roche Applied Science, Indianapolis, IN, USA), as previously described [35]. Briefly, cardiomyocytes were fixed with 4% paraformaldehyde in PBS for 40 min at room temperature, washed and permeabilized with 0.1%Triton X-100 for another 5 min at 4 °C. The fixed cells were subjected to TUNEL staining according to the manufacturer’s instructions. Finally, the cells were incubated with 4′,6-diamidino-2-phenylindole (DAPI) for 15 min. Fluorescence images of ten random fields from each sample were taken using a fluorescence microscope.

### 4.7. Estimation of Oxidative Stress Biomarker Levels in Cardiac Tissues, Myocytes and Fibroblasts

Cardiac tissue and neonatal rat cardiomyocytes and fibroblasts were harvested and lysed in lysis buffer (phosphate-buffered saline (PBS), 1% NP-40, 0.5% sodium deoxycholate, and 0.1% sodium dodecyl sulfate (SDS)) containing 1 mM phenylmethylsulfonyl fluoride (PMSF) and incubated for 30 min on ice. After centrifugation at 4 °C and 12,000× *g* for 15 min, the supernatant was collected for protein quantification. MDA content and SOD activity were determined following the instructions of the lipid peroxidation assay kit and enzymatic activity assay kit. Cardiac myocytes and fibroblasts were collected, and ROS production was quantified using 2′,7′-dichlorodihydrofluorescein diacetate and analyzed by flow cytometry at an excitation wavelength of 495 nm and emission wavelength of 520 nm.

### 4.8. Determination of Mitochondrial Morphological Changes and the Mitochondrial Membrane Potential

Cardiomyocytes were collected by centrifugation at 4 °C and 100× *g* for 5 min. Glutaraldehyde was added to the cells, followed by centrifugation at 4 °C and 200× *g* for 5 min and addition of 1% osmium tetroxide for 90 min. PBS (0.1 M) was used to wash the cells after centrifugation, followed by incubation with 50, 70, and 90% ethanol, 90% acetone/90% ethanol (1:1), and 90% acetone for 15 min. After centrifugation, the cells were washed three times with 100% acetone for 10 min each and subsequently treated with 100% acetone/embedding agent (1:1) overnight, embedding agent in a desiccator for 6 h, and 2,4,6-tris-(dimethylaminomethyl) phenol (DMP-30)-containing embedding agent in an oven at 40 °C for 3–5 days. The embedded samples were trimmed into a shape suitable for ultrathin slicing to observe the mitochondrial morphology using transmission electron microscopy (TEM).

The mitochondrial membrane potential in cardiomyocytes and fibroblasts was assessed by 5,5′,6,6′-tetrachloro-1,1′,3,3′-tetraethylbenzimidazolcarbocyanine iodide (JC-1) staining and a laser scanning confocal microscope as described previously [35]. JC-1 can accumulate and aggregate (red fluorescence) in normal mitochondria. Loss of the mitochondrial membrane potential prevents JC-1 entry into mitochondria, and monomeric JC-1 (green fluorescence) remains in the cytosol. Cardiomyocytes and fibroblasts from different treatment groups were washed, incubated with JC-1 at 37 °C for 15 min, washed again and mounted on a Leica fluorescence microscope for imaging. The ratio of the aggregated JC-1 fluorescence intensity to monomeric JC-1fluorescence intensity was used to quantify changes in the mitochondrial membrane potential.

### 4.9. Extraction of Protein from Cardiac Tissue and Cardiomyocytes

Cardiomyocytes, cardiac tissues and fibroblasts were lysed in lysis buffer containing 1 mM PMSF by incubation for 30 min on ice and then centrifuged at 4 °C and 12,000× *g* for 15 min. The supernatant lysates were diluted in 2× or 5× SDS sample buffer and boiled for 8 min. Unless specified, whole-cell or tissue lysates were used for analysis. Mitochondrial and nuclear proteins were isolated using a mitochondria isolation kit (Thermo Fisher Scientific, Rockford, IL, USA) and NE-PER™ nuclear/cytoplasmic extraction reagent (Thermo Fisher Scientific, Rockford, IL, USA), respectively, according to the manufacturer’s instructions.

### 4.10. Western Blot Analysis

The samples were resolved on an SDS-polyacrylamide gel and then transferred to polyvinylidene fluoride membranes (Millipore, Billerica, MA, USA) or nitrocellulose membranes (Millipore, Billerica, MA, USA). The membranes were blocked in tris-(hydroxymethyl) aminomethane (Tris)-buffered saline containing 5% bovine serum albumin (20 mM Tris-HCl, 137 mM NaCl, and 0.1% Tween 20) for 1 h at room temperature. The membranes were subsequently incubated overnight at 4 °C with primary antibodies. Following incubation with horseradish peroxidase-conjugated secondary antibodies, the immunoblots were exposed to film using enhanced chemiluminescence reagents (Thermo Fisher Scientific, Rockford, IL, USA). ImageJ software (v1.53), an open-source image-processing program, was used to quantify the band density.

### 4.11. Immunofluorescence Staining

The cardiac fibroblasts were fixed with 4% formalin solution and then treated with 0.1% Triton X-100 in PBS. After 3 washes with PBS for 5 min each time, the cells were blocked in PBS with 1% BSA, followed by incubation with a 1:200 dilution of antibodies against α-SMA and vimentin at 4 °C overnight. The cells were then incubated with the secondary antibodies Alexa Fluor 488-labeled goat antibody against mouse IgG and Alexa Fluor 647-labeled goat antibody against rabbit IgG (1:400 dilution) for 1 h. Finally, after washing three times, the cells were incubated with a 1:200 dilution of DAPI for 15 min and observed via fluorescence microscopy.

### 4.12. Statistical Analyses

Data are expressed as the mean ± standard error of the mean (SEM). Statistical differences among groups were evaluated using one-way analysis of variance followed by Bonferroni post hoc analysis. Differences were considered statistically significant at *p* < 0.05. Data were analyzed using SPSS software (Version 13.0, Chicago, IL, USA).

## 5. Conclusions

In conclusion, the present study demonstrated for the first time that Ber prevents cardiac diastolic dysfunction and fibrosis in DOX-challenged rats by alleviating cardiomyocyte injury and CF differentiation. The molecular mechanism involves Ber-mediated activation of the Nrf-2 pathway, which attenuates DOX-induced oxidative stress and mitochondrial damage in both cardiomyocytes and cardiac fibroblasts. Considering that our previous study proved that Ber suppresses DOX-induced cardiomyocyte apoptosis, we suggest that Ber may be a promising therapeutic adjuvant that protects the heart from the serious side effects of DOX.

## Data Availability

The data that support the findings of this study are openly available on request to the corresponding author.
